# Headwaters are critical reservoirs of microbial diversity for fluvial networks

**DOI:** 10.1098/rspb.2013.1760

**Published:** 2013-11-22

**Authors:** Katharina Besemer, Gabriel Singer, Christopher Quince, Enrico Bertuzzo, William Sloan, Tom J. Battin

**Affiliations:** 1 Department of Limnology and Oceanography, University of Vienna, Althanstrasse 14, 1090 Vienna, Austria; 2 WasserCluster Lunz GmbH, Dr Carl Kupelwieser Promenade 5, 3293 Lunz am See, Austria; 3 School of Engineering, University of Glasgow, Glasgow G12 8QQ, UK; 4 Laboratory of Ecohydrology, School of Architecture, Civil and Environmental Engineering, Ecole Polytechnique Fédérale Lausanne, 1015 Lausanne, Switzerland

**Keywords:** microbial biodiversity, biofilms, fluvial networks, alpha diversity, beta diversity, headwaters

## Abstract

Streams and rivers form conspicuous networks on the Earth and are among nature's most effective integrators. Their dendritic structure reaches into the terrestrial landscape and accumulates water and sediment en route from abundant headwater streams to a single river mouth. The prevailing view over the last decades has been that biological diversity also accumulates downstream. Here, we show that this pattern does not hold for fluvial biofilms, which are the dominant mode of microbial life in streams and rivers and which fulfil critical ecosystem functions therein. Using 454 pyrosequencing on benthic biofilms from 114 streams, we found that microbial diversity decreased from headwaters downstream and especially at confluences. We suggest that the local environment and biotic interactions may modify the influence of metacommunity connectivity on local biofilm biodiversity throughout the network. In addition, there was a high degree of variability in species composition among headwater streams that could not be explained by geographical distance between catchments. This suggests that the dendritic nature of fluvial networks constrains the distributional patterns of microbial diversity similar to that of animals. Our observations highlight the contributions that headwaters make in the maintenance of microbial biodiversity in fluvial networks.

## 1. Introduction

A major focus of ecology has been to understand the processes that shape biodiversity at local and landscape level [1,2]. Only recently have theoretical ecologists begun to explore and predict patterns of biodiversity in dendritic landscapes [3–5], among which fluvial networks are prominent examples. It has been shown that the effective one-dimensional dispersal imposed by a dendritic network enhances biodiversity over-and-above that which would emerge in a two-dimensional landscape; hence, the areally averaged biodiversity will be greater in fluvial networks than in the oceans [4].

In stream and river ecology, biodiversity patterns have traditionally been studied along the longitudinal continuum that these ecosystems form—as epitomized by the river continuum concept (RCC) [6]. This concept emphasizes downstream environmental and ecological changes and predicts that biodiversity peaks in mid-sized streams, where environmental heterogeneity is assumedly highest. Studies on fish [7] and invertebrates [8,9] support the view of increasing local diversity (i.e. alpha diversity) from headwaters to mid-sized streams.

However, streams and rivers not only form a longitudinal continuum, but they also form fluvial networks [10], whose dendritic nature may have implications for biodiversity patterns beyond purely longitudinal constraints. The high abundance of headwaters and their position at the tips of a fluvial network indicate that a substantial part of network-wide biodiversity may rest in the spatial variation of community composition among streams, that is, beta diversity [8]. Work on invertebrate communities suggests that headwaters exhibit high beta diversity compared with mid-sized steams [8], an observation that is supported by experimental work with protozoan metacommunities [11]. These patterns may be attributable to large environmental variation among headwaters [12], their spatial isolation limiting dispersal [11] and their high abundance within fluvial networks [13,14].

Furthermore, stream confluences, as conspicuous nodes in the fluvial network, have been postulated to augment biodiversity of a network by way of accumulating species from multiple catchments and, thus, increasing the size of the metacommunity from which local communities assemble [5,11,12]. As posited by the network dynamics hypothesis, strong gradients of channel geomorphology across confluences may also increase habitat heterogeneity and community variation, which would have a knock-on effect on network scale biodiversity [15]. However, empirical observations supporting these conjectures are sparse. Field studies have revealed elevated fish diversity around confluences [16,17] and, similarly, laboratory work on protozoan metacommunities evoked that dispersal increases diversity in experimental confluences characterized by higher connectivity [11].

In streams and rivers, microbial life is dominated by benthic biofilms, which control key ecosystem processes [10]. The biodiversity of these biofilms results from the interplay of local environmental conditions and the dispersal dynamics of microorganisms from the source community suspended in the streamwater [18]. Microorganisms are primarily passive dispersers [19]; the directionality of the water flow generating asymmetrical dispersal, together with the dendritic network structure, are therefore likely to influence microbial diversity patterns [20]. Understanding microbial biodiversity patterns at the scale of entire fluvial networks is of paramount importance, especially since headwaters are increasingly under threat by burial, mountain-top mining and inter-basin water transfer [21,22].

In this study, we investigated patterns of microbial alpha and beta diversity in benthic biofilms throughout a fluvial network. We leaned on the concept of metacommunity (i.e. a set of local communities linked by dispersal) ecology [2] to guide our understanding of microbial diversity. Specifically, we predicted higher alpha diversity downstream than upstream of confluences because of increasing metacommunity size [23]. Furthermore, based on the converging structure of fluvial networks [7], we hypothesized that microbial alpha diversity increases from headwaters downstream, a pattern that may be amplified by significant downstream dispersal of small organisms with water flow [11]. We also predicted that microbial beta diversity decreases from headwaters downstream because of dispersal limitations [11] and pronounced habitat variation among headwaters [12].

## 2. Material and methods

### (a) Study area and field survey

**Figure 1.**
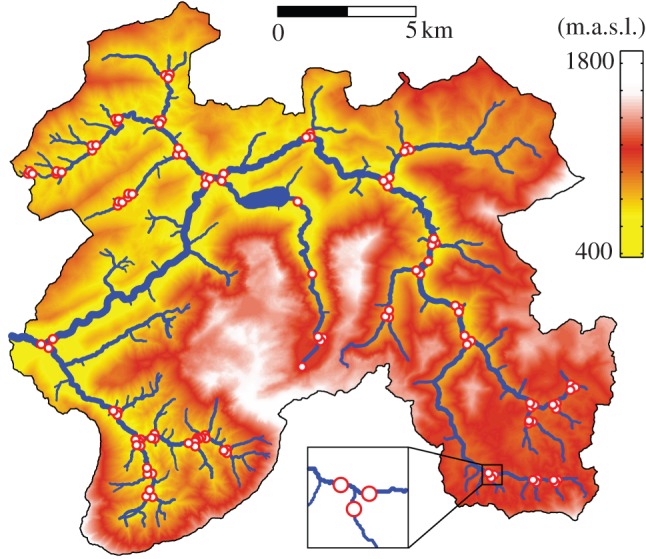
The network of the River Ybbs upstream of Göstling, Austria. Red circles indicate sampling sites (*n* = 114). Most sampling sites (*n* = 102) were located immediately upstream or downstream of a confluence, as depicted in the insert. The catchment area is colour-coded according to elevation (m.a.s.l.).

We sampled benthic biofilms from 114 streams within a pre-alpine catchment (River Ybbs, Austria; 254 km^2^; 1893–532 metres above sea level (m.a.s.l.); figure 1). Catchment geology is dominated by dolomite (82%) and karst; forests (82%) and alpine meadows (11%), characterized land use, bare rock, agricultural areas and settlements constitute minor parts of the catchment (7% in total). Streams were sampled during a one-week period in winter after prolonged baseflow. This was to ensure rather stable and homogeneous hydrological conditions throughout the fluvial network and to sample mature biofilms with reduced successional dynamics [24]. Discharge ranged from less than 1 l s^−1^ in the smallest headwaters to 2282 l s^−1^ in the fifth-order stream during the survey. To assess the relevance of confluences for biodiversity patterns, we primarily sampled tributary pairs upstream of their confluence and the recipient streams downstream of their confluence (figure 1). Recipient streams were sampled 10–20 times the channel width or at least three riffle-pool sequences downstream of the confluence [25] to ensure complete mixing of the streamwater, while retaining the characteristics of the confluence environment [15]. Mixing of streamwater was confirmed by measuring electrical conductivity. Sampling was primarily designed to cover important confluences, while equally representing all orders and sizes of streams. A number of additional samples were taken at the inflow and outflow of lakes to complete the picture of the network.

Stream channel depth, width, slope, velocity and discharge were measured in the field following standard procedures. The dimensionless Froude number was calculated as an integrative descriptor of streambed hydraulics [14]. A digital elevation model, rigorously ground-truthed, served to compute network metrics, hydrologic distances between sampling sites, the size of sub-catchments and land use (see the electronic supplementary material, methods). Streamwater was analysed for NO_3_, NH_4_ and PO_4_ concentrations and dissolved organic matter (DOM) was characterized using fluorescence and spectrophotometric techniques (electronic supplementary material, methods). From each site, 6–12 stones (1–4 cm in diameter) were sampled over a horizontal transect and stored in sterile tubes pending further processing.

### (b) DNA extraction, PCR amplification and 454 pyrosequencing

In the laboratory, microbial biomass was removed from the stones using sterile tweezers and spatulas and DNA was extracted using the PowerSoil DNA Isolation Kit (MoBio, Carlsbad, CA, USA) following the manufacturer's recommendations. The V4 and V5 regions of the 16S rRNA gene were amplified using the primers 515F 5′-GTGNCAGCMGCCGCGGTAA-3′ and 926R 5′-CCGYCAATTYMTTTRAGTTT-3′ (Invitrogen, Vienna, Austria) [26]. To reduce potential PCR bias generated by multiplex identifiers, we used a 2-step PCR [27] (see the electronic supplementary material, methods). Equal amounts of the barcoded PCR-products were mixed and submitted to the Centre for Genomic Research (Liverpool, UK) for pyrosequencing on a 454 GS20 FLX Titanium platform. Pyrosequencing data were cleaned using the software package AmpliconNoise v. 1.21 [26]. The cleaned reads were clustered to operational taxonomic units (OTUs) with a complete linkage algorithm on a 97% sequence identity level, yielding a clean dataset of 1 502 594 reads constituting 14 407 OTUs. The sequence data have been submitted to the NCBI Sequence Read Archive under accession number SRX344129.

### (c) Data analysis

For the estimation of alpha diversity and evenness, we employed a range of indices, which differentially weight abundant and rare species. Namely, we used richness, the number equivalents of the Shannon entropy (i.e. Shannon diversity) and of the Gini-Simpson coefficient (i.e. Simpson diversity), which might be interpreted as the number of all species, of common species and of abundant species [28]. The relative logarithmic evenness was calculated from OTU richness and Shannon diversity (RLE_0,1_, equivalent to Pielou evenness) and from Shannon and Simpson diversities (RLE_1,2_) [28]. We took this approach of diversity and evenness estimation to circumvent problems associated with the under-sampling bias inherent in microbial field data [29]. Richness is most impacted by under-sampling, whereas high-level diversity estimates (e.g. Simpson diversity) are robust against under-sampling but omit information included in the dataset; the same is true for the respective evenness measures [28]. To account for differences in sequencing effort, all communities were rarefied to the lowest number of reads obtained from an individual sample (4698) prior to analysis (see the electronic supplementary material, methods).

Changes of alpha diversity, evenness, Froude number, water depth, water velocity and channel slope at each confluence were calculated as the average of the tributary pairs and compared to the respective recipient streams using a Wilcoxon test for paired samples. The differences between the alpha diversity and evenness upstream and downstream of the confluences were then tested for correlation with the changes of the physical parameters using Spearman's rank correlation. Only confluences for which a sample triplet (consisting of two tributaries and one recipient stream) existed were included in this analysis (102 samples representing 34 confluences).

To study diversity distribution at the level of the fluvial network, we plotted the various indices of alpha diversity and evenness against the logarithm of the catchment size. Owing to decreasing dispersion of the data with catchment size, we applied semi-parametric regression type models (Generalized Additive Models for Location, Scale and Shape, GAMLSS), which allow modelling of not only the mean (location), but also the dispersion (scale) and shape of the distribution of the response variable [30] (see the electronic supplementary material, methods).

We divided the OTU matrix into taxa that are regionally common (i.e. core taxa) and taxa with occasional occurrence (i.e. satellite taxa). OTUs occurring in more than or equal to 50% of the samples were regarded as core OTUs, all others as satellite OTUs. Their relative importance for each community was estimated as the percentage of the total number of reads affiliated to core- and satellite OTUs, respectively, in each sample, and data were regressed on the logarithm of the catchment size.

We assessed the explanatory value of environmental variables using a forward selection procedure. Environmental variables included network and land-use descriptors, stream geomorphological and hydraulic parameters, and streamwater chemistry such as DOM properties and nutrients. Visual inspection of the data indicated bivariate relationships between the proportion of reads classified as cyanobacteria (excluding chloroplasts) and both biofilm diversity and evenness. We therefore included the relative abundance of cyanobacteria as a possible biological control in the forward selection procedure (electronic supplementary material, methods). Variables that explained most of the variation were identified using forward selection [31]. To estimate the direction of these relationships, multiple regression analysis was performed to calculate the partial standardized regression coefficients for the variables retained by the forward selection procedure. Spearman's rank correlation analysis was performed to test for relationships among environmental variables.

To explore the spatial turnover (i.e. beta diversity) of OTUs among small headwaters and mid-sized streams, we compared the distance decay of similarity of these two groups. For this, sampling sites were grouped by a threshold criterion defined by Strahler stream order and catchment size, because the Strahler order does not necessarily reflect stream size [32]. Catchments smaller than the largest catchment of a sampled first-order catchment (*ca* 5 km^2^) were classified as headwaters (first to third stream order, *n* = 50), all others as mid-sized streams (second to fifth stream order, *n* = 64). We employed the Sørensen, Horn and Morisita-Horn indices of pairwise community overlap, which differ in their sensitivity towards rare species. Equivalent to the estimation of alpha diversity, communities were randomly resampled to 4698 reads before similarity between a sample pair was calculated; each entry in the similarity matrices was then calculated as the average of 1000 such similarity values (see the electronic supplementary material, methods). Similarities between flow-connected pairs were excluded from the analysis to avoid inflation of similarity between mid-sized streams because of the higher degree of flow connectivity among these [8,33].

Distance decay curves for headwaters and mid-order streams were calculated by fitting linear models to the decline of similarity with increasing hydrologic distance. Using analysis of covariance (ANCOVA), the effect of group affiliation on the similarity between sample pairs was tested while controlling for the effect of distance as a covariate. Accounting for distance was necessary as first-order streams are likely to be further apart from each other than higher order streams [13], which may result in a greater range of habitat conditions. We used hydrologic distance, to account for environmental variables that are related to the fluvial network (e.g. presence of actively dispersing grazers), and Euclidean distance, representing spatially auto-correlated environmental variables at the catchment scale (e.g. geology, land use). Using pairwise community similarity implies non-independence in the values of the dependent variable; we therefore computed significances for both model terms from null distributions of the respective *F*-values built from 999 random permutations of community composition data among sites using functions of the R-package lmPerm [34]. The probability density distributions of the similarity values from each group were computed using a Gaussian kernel with a bandwidth given by the Normal Reference Rule [35]. The program R v. 2.13.0 [36] was used for all data analyses.

## 3. Results

**Figure 2.**
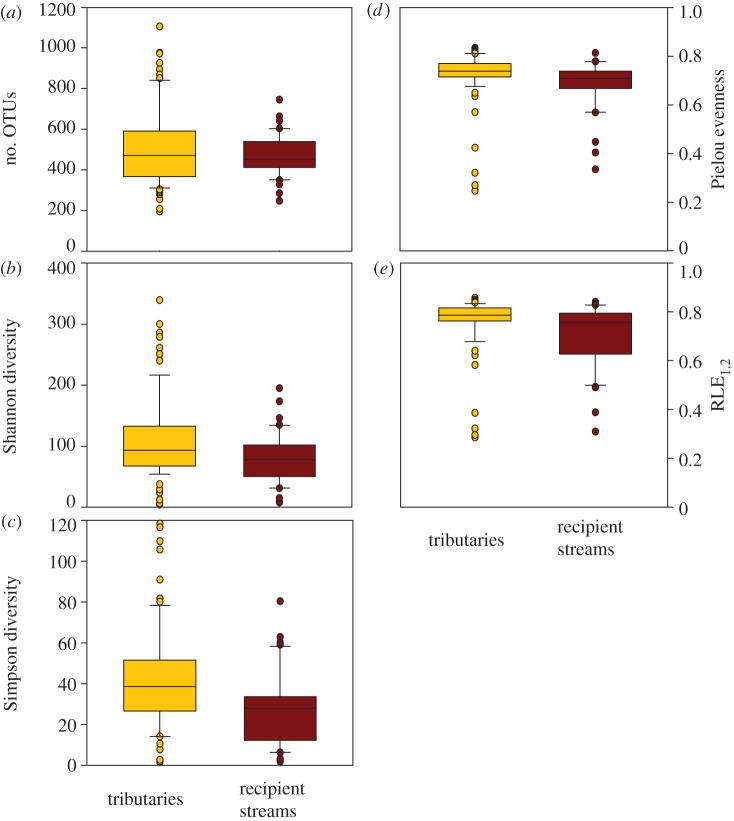
Biofilm diversity and evenness at confluences. (*a*) Mean richness, (*b*) Shannon diversity, (*c*) Simpson diversity, (*d*) Pielou evenness and (*e*) RLE_1,2_, upstream (tributaries, yellow) and downstream (recipient streams, red) of confluences. Recipient streams (*n* = 34) had significantly lower Shannon diversity (*p* < 0.05), Simpson diversity (*p* < 0.05), Pielou evenness (*p* < 0.01) and RLE_1,2_ (*p* < 0.05); OTU richness did not differ significantly.

Alpha diversity, expressed as Shannon and Simpson diversity, was significantly lower in biofilms downstream than upstream of the confluences; average OTU richness did not differ significantly (figure 2). Both evenness measures were also significantly lower downstream than upstream of the confluences (figure 2). To test whether these diversity patterns were attributable to hydromorphological shifts across confluences, we evaluated changes in the Froude number, water depth, water velocity and channel slope, respectively. Although depth, velocity and channel slope varied significantly (*p* < 0.001, *n* = 34) between tributaries and recipient streams, none of these variables explained the observed shifts in microbial diversity and evenness across confluences.

**Figure 3.**
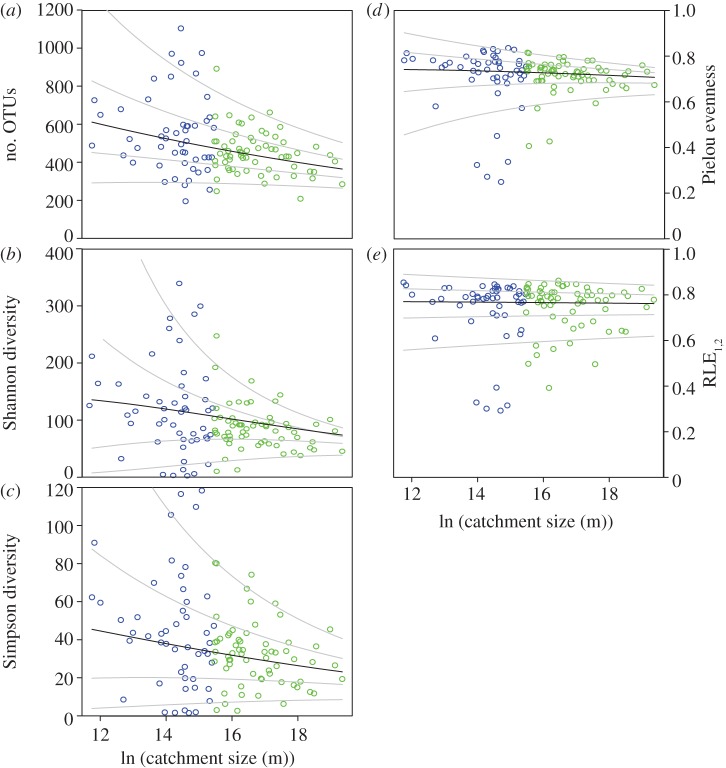
Biofilm diversity and evenness across the River Ybbs network. (*a*) Richness, (*b*) Shannon diversity, (*c*) Simpson diversity, (*d*) Pielou evenness and (*e*) RLE_1,2_, plotted against the logarithm of the catchment size (*n* = 114). The black line represents the fitted GAMLSS, the grey lines are percentile curves for 5, 25, 75 and 95% of the data. Location (mean) and scale (dispersion) of richness, Shannon diversity, Simpson diversity and Pielou evenness decreased significantly with catchment size (location: *p* < 0.001 for richness and Shannon diversity, *p* < 0.01 for Simpson diversity and Pielou evenness; scale: *p* < 0.05 for richness and Simpson diversity, *p* < 0.001 for Shannon diversity and Pielou evenness). RLE_1,2_ showed no trend. For consistency with figure 4, headwaters (less than 5 km^2^) are displayed blue, mid-sized streams green.

At the scale of the study network, richness, Shannon diversity and Simpson diversity all exhibited a decreasing trend from small to large streams; also, the variability of these indices decreased markedly downstream. For instance, richness ranged from 196 to 1106 OTUs in small streams (catchment size less than 5 km^2^) and became constrained between 209 and 483 OTUs in larger streams (catchment size more than 71 km^2^). GAMLSS analyses [30] revealed that these downstream patterns of alpha diversity were significant for both the mean and the variance (figure 3). Similarly, both the mean and variance of the Pielou evenness declined downstream, yet not as clearly as for diversity. RLE_1,2_ did not show any downstream trend (figure 3).

To gain further insight into alpha diversity patterns, we separated the OTU matrix into core and satellite taxa, yielding 330 core OTUs and 14 077 satellite OTUs. Core OTUs contributed 81±13% (mean±s.d.) to the total number of reads per sample and their proportion showed an increasing trend with catchment size; accordingly, the contribution of satellite OTUs decreased with catchment size (see the electronic supplementary material, figure S1).

Forward variable selection of environmental variables [31] revealed that specific ultraviolet absorbtion at 254 nm (SUVA_254_), a proxy for streamwater DOM aromaticity [37], and the relative abundance of cyanobacteria in the biofilm samples explained most of the variance of diversity and evenness observed at network level (table 1). Multiple regression analysis further showed that diversity and evenness were positively related to SUVA_254_ and negatively to the relative abundance of cyanobacteria. Moreover, catchment size, the position relative to a confluence (tributary versus recipient stream), channel slope, forest cover, the Froude number and the concentration of dissolved organic carbon (DOC) contributed to the explained variance (table 1). Although the explanatory variables were checked for multi-colinearity prior to analysis, correlation analyses revealed a significant decrease of SUVA_254_ (*R* = −0.36, *p* < 0.001) and channel slope (*R* = −0.61, *p* < 0.001), and a significant increase of the Froude number (*R* = 0.42, *p* < 0.001) with catchment size of the study streams; DOC concentration showed no significant change downstream. The relative abundance of cyanobacteria was not significantly correlated to catchment size or to any of the hydrological or geomorphological parameters.

**Table 1.** Environmental variables explaining the variation of diversity and evenness measures, as retained by a forward selection procedure. (*R*^2^ is the coefficient of multiple determination for all consecutively added variables calculated using an alpha significance level of *p* < 0.05. Here *b* is the partial standardized regression coefficient obtained by multiple regression including all retained variables and shows the direction of the relationship.)

richness	*R*^2^	*b*	Shannon diversity	*R*^2^	*b*	Simpson diversity	*R*^2^	*b*	Pielou evenness	*R*^2^	*b*	RLE_1,2_	*R*^2^	*b*
SUVA_254_	0.12	0.31	cyanobacteria	0.24	−0.56	cyanobacteria	0.27	−0.57	cyanobacteria	0.65	−0.85	cyanobacteria	0.67	−0.87
cyanobacteria	0.26	−0.39	SUVA_254_	0.37	0.28	SUVA_254_	0.39	0.29	SUVA_254_	0.68	0.16	position to confluence^a^	0.69	−0.14
catchment size	0.31	−0.24	catchment size	0.41	−0.14	channel slope	0.44	0.26	Froude number	0.71	−0.16	Froude number	0.71	−0.13
			channel slope	0.43	0.21	forest cover	0.47	0.20				DOC	0.72	0.11
			forest cover	0.46	0.19	position to confluence^a^	0.49	−0.17						

^a^‘Position to confluence’ was coded as binary variable: tributary = 0, recipient stream = 1, therefore a negative relation indicates lower diversity/evenness in the recipient stream.

**Figure 4.**
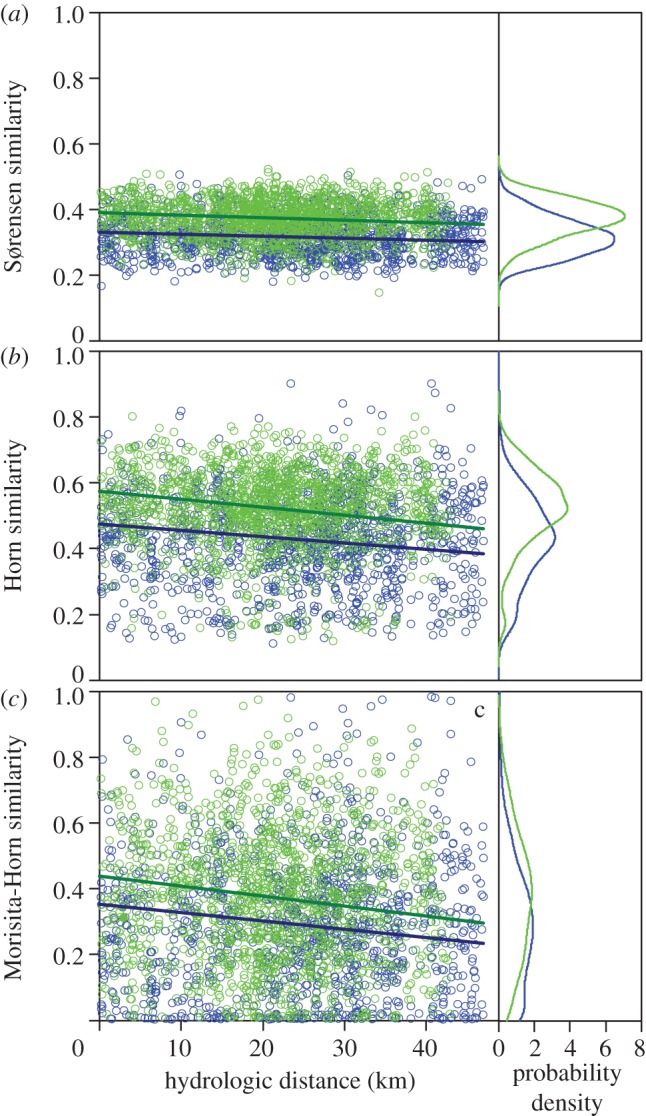
Distance decay of the (*a*) Sørensen, (*b*) Horn and (*c*) Morisita-Horn similarities with increasing hydrologic distance (left panel). ANCOVA revealed significantly (*p* < 0.001) lower similarities between headwaters (blue, *n* = 1184) than between mid-sized streams (green, *n* = 1475), but no significant decline of similarity with distance. The right panel displays the probability density distribution of the similarity values.

The ANCOVA showed significantly lower Sørensen, Horn and Morisita-Horn similarities among headwaters than among mid-sized streams for both hydrologic and Euclidean distance (*p* < 0.001 for both distance types and all three similarity indices), indicating higher beta diversity in headwaters. The similarity decay with the hydrologic or Euclidean distance was not significant (figure 4; electronic supplementary material, figure S2).

## 4. Discussion

Our study reveals headwaters as critical reservoirs for microbial diversity in fluvial networks. Mean microbial alpha diversity decreased from headwaters downstream, which contrasts predictions of the RCC [6] and patterns reported from studies on invertebrate [8,9] and fish [7,16,17] assemblages. Our initial expectation rested on the assumption that biofilm communities downstream of confluences recruit downstream-dispersing propagules from both catchments upstream [5,12], thereby increasing alpha diversity in downstream direction. Unexpectedly, confluences even tended to reduce alpha diversity. The differing results obtained from the various diversity indices indicate that this reduction was attributable to decreasing numbers of abundant OTUs as supported by the significant drop in evenness. Our results suggest that the local environment and biotic interactions may modify the influence of metacommunity connectivity on local biofilm biodiversity throughout the network [5,23].

To explain the unexpected pattern of alpha diversity at network scale, we resort to principles inherent to streams across fluvial networks. Headwaters are intimately connected with the terrestrial environment [38,39] and are characterized by a large ratio of benthic surface area to water volume, relative to larger fluvial ecosystems downstream [10]. Therefore, we suggest that headwaters collect microorganisms from terrestrial sources [40], which can contribute to community assembly of benthic biofilms [18]. This notion is supported by elevated values of SUVA_254_ in headwaters and its positive correlation with alpha diversity. As a measure of aromaticity [37], SUVA_254_ typically points to terrestrial contributions to streamwater DOM, suggesting common terrestrial sources of microbes and DOM. We recognize that microbial taxa also enter mid-order streams laterally and via shallow groundwater flow paths. However, this effect probably becomes alleviated moving downstream, because of changing hydraulic geometry of stream channels [10,41]. Alternatively, the relationship between alpha diversity and SUVA_254_ may point to DOM composition as a control of microbial diversity. Indeed, as a consequence of lateral terrestrial inputs, headwaters have been proposed to contain the highest organic matter diversity in stream ecosystems [6,42]; however, evidence for this assumption is still lacking. Additional environmental control of alpha diversity may be exerted by hydromorphological variables such as slope and Froude number.

The pronounced variability of alpha diversity among the smaller streams in this study is in line with patterns found in experimental protozoan metacommunities [11]. We therefore suggest that the spatial variance of alpha diversity bears an imprint of a dispersal limitation effect in the headwaters of a fluvial network. Additionally, increased variability of alpha diversity among headwaters may reflect higher habitat and resource variation in headwaters [12] or various degrees of microbial immigration from adjacent soils because of locally divergent hydrological flow paths [14].

Confluences can be sites of abrupt changes in geomorphology, potentially supporting distinct biotic communities in the tributaries and the recipient stream [15,25]. As streambed geomorphology can influence benthic biofilm communities [24,43], we tested whether physical changes (e.g. water depth, velocity, channel slope or Froude number) explain the observed drop in alpha diversity and evenness across confluences. The fact that we did not find any relationship is notable given that channel slope and Froude number were among the candidate variables explaining diversity and evenness, respectively, at network scale, indicative of their potential influence on biodiversity patterns at the larger scale.

Competition can reduce alpha diversity and evenness [44]. We speculate therefore that biotic interactions contribute to the observed drop in alpha diversity and evenness downstream of confluences. Indeed, high metacommunity connectivity can have negative effects on local diversity and evenness by amplifying species competition [45], and the larger regional species pool downstream of confluences is more likely to include taxa better adapted to the streambed environment. Cascading through the network, the accumulation of potentially superior competitors could ultimately decrease mean alpha diversity. This notion is supported by our observation that the relative importance of core OTUs increased, whereas that of satellite OTUs decreased downstream. A recent study [40] showing decreasing alpha diversity and evenness from upslope soils to headwaters and downstream lakes corroborates this assumption.

The relationship between the relative abundance of cyanobacteria and the overall diversity and evenness of biofilm communities further underscores the potential role of biotic interactions shaping patterns of biofilm biodiversity. Cyanobacteria can be abundant components of benthic biofilms [43] and they are well known for allelopathy as a competitive strategy [46]. The spatial variation of cyanobacterial abundance throughout our study streams may therefore further contribute to the network scale pattern of alpha diversity. We did not find any relationship between the relative abundance of cyanobacteria and any of the hydraulic parameters and therefore exclude confounding effects.

The lower similarity of biofilm communities among headwaters than among mid-sized streams supports our hypothesis of high beta diversity in headwaters in a fluvial network. This is in accordance with earlier studies on invertebrates [8,13], but the underlying mechanisms remain debated [8,13,47]. Headwaters are generally more isolated from each other than larger streams, evoking dispersal limitation to potentially enhance beta diversity among these systems [8,11,12]. Headwaters also encompass a larger geographical area compared with downstream catchments, potentially resulting in a wider range of environmental conditions that biota experience in these systems [13,47]. As the ANCOVA controlled for a possible distance effect, the higher beta diversity found in headwaters compared with mid-sized streams cannot be explained exclusively by the larger geographical distance among headwaters and, hence, by spatially auto-correlated environmental variables. This suggests that the dendritic nature of fluvial networks constrains microbial dispersal and leads to elevated beta diversity between headwaters [8,11]. This notion of dispersal limitation is in line with the alpha diversity patterns probably imprinted by terrestrial microorganisms in headwaters and preferential downstream dispersal of core OTUs.

To achieve comparability between the headwaters and mid-sized streams, we removed all similarities between flow-connected sites [8,33]. Therefore, the community pairs were not connected by downstream dispersal via water flow. Any distance decay of similarity would therefore be caused by spatially auto-correlated environmental variables or dispersal, which is not restricted to passive downstream dispersal. Although we found no significant distance decay of similarity, decreasing trends of the Horn and the Morisita-Horn indices suggest that spatial auto-correlation may occur at larger spatial scales.

Research on animal biodiversity has taught us that high beta diversity of headwater communities makes these communities critical for regional diversity and its conservation [8,13,21]. Our study expands this insight now to the microbial realm. The fact that both alpha and beta diversity is higher in headwaters underscores the relevance of the smallest streams in a fluvial network as reservoirs for downstream microbial biodiversity. Given the global deterioration and loss of headwaters [22], our findings have broad consequences for the conservation and management of microbial diversity in fluvial networks and for the ecosystem functions and services they provide.

## Data accessibility

The sequence data are available at the NCBI Sequence Read Archive under accession number SRX344129.
